# Reply to: underestimating the impact of erect abdominal radiographs

**DOI:** 10.1002/jmrs.333

**Published:** 2019-04-10

**Authors:** Ashish Chawla, Wilfred C. G. Peh

**Affiliations:** ^1^ Department of Diagnostic Radiology Khoo Teck Puat Hospital Singapore Singapore; ^2^ Department of Diagnostic Radiology Khoo Teck Puat Hospital Singapore Singapore

We thank Snaith and Flintham for their comments on our editorial^1^ where we emphasised the judicious use of abdominal radiographs, with a major concern being additional radiation from an unnecessary erect radiograph, and mentioned that performing two abdominal radiographs implied a doubling of radiation dose. We are grateful to Snaith and Flintham for highlighting their work which further supports the point that we were making. Although we could not find the actual radiation dosages in the quoted pilot study^2^ evaluating the differences in patient body habitus between erect and supine radiographs, we agree that there should be logically a difference in the radiation dose between the two types of radiographs resulting from changes in soft tissue thickness due to removal of compressive forces, gravity and organ repositioning. We look forward to the publication of their study which we feel will be an important addition to the literature.

## Conflict of Interest

The authors declare no conflict of interest.
